# Supplementary material for: Modelling the spatiotemporal dynamics of senescent cells in wound healing, chronic wounds, and fibrosis
Source: PLoS Comput Biol. 2025 Apr 15;21(4):e1012298. doi: 10.1371/journal.pcbi.1012298 (PMC12052216; doi:10.1371/journal.pcbi.1012298)
Supplement: S1 Text — Supplementary methods, Supplementary results, Supplementary figures (Fig A Data used for PSO, Fig B-H Wound healing model multidimensional sensitivity analysis), Supplementary tables (Table A Baseline parameter values and conversion factors for the wound healing model, Table B Summary of PSO output for individual swarms, Table C Summary of results from PSO). (DOCX) [file pcbi.1012298.s001.docx]

# Supplementary

## Supplementary methods

In the Cellular Potts Model (CPM) framework, the simulation lattice is divided into collections of pixels representing generalised ‘cells’, each with an assigned ‘cell’ type. ‘Cells’ interact with each other and their environment based on parameters that can approximate biological constraints. These parameters can be based on their in vitro or in vivo biological counterparts and steer the simulation. The CPM evolves through time using an effective energy Hamiltonian (H) and Boltzmann Factor acceptance function that is calculated at each simulation time step (a Monte-Carlo step, MCS). The Boltzmann Factor uses a Potts “Cell membrane fluctuation amplitude” (Tm) that allows the simulated cells to explore an energetically plausible trajectory through time instead of simply minimizing the calculated system energy, which tends to get trapped in local energy minima. Terms of the effective energy equation for the wound healing model will be described in the next sections.

### Cell dynamics and motility

Before every attempt, a source pixel $\vec{i}$ and a target pixel $\vec{i} '$ are chosen. If $\sigma_{\vec{i}}$and $\sigma_{\vec{i}'}$belong to different generalised ‘cells’, then the difference in energy $\left( \Delta H \right)$ to copy the index (unique ID of a pixel) from the source to the target site is calculated. With that, the probability of accepting a new model configuration is given by:

|  | $P\left( \sigma_{\vec{i}}\to\sigma_{\vec{i}'} \right)= \left\{ \begin{aligned} 1, for \Delta H \leq0 \\ f\left( \Delta H \right), for \Delta H >0 \end{aligned} \right.$ | 1 |
| --- | --- | --- |

Where,

|  | $f\left( \Delta H \right)= e^{\frac{-\Delta H}{T_{m}}}$ | 2 |
| --- | --- | --- |

Where the parameter $T_{m}$ represents the amplitude of cell membrane fluctuations in the model. This means that the new configuration (from $\sigma_{\vec{i}}$to $\sigma_{\vec{i}'}$) is accepted if the difference in effective energy calculated between the two configurations is less than or equal to zero. Otherwise, a new probability for the change in model configuration from $\sigma_{\vec{i}}$to $\sigma_{\vec{i}'}$ is calculated using equation 2. The overall aim of this algorithm is explore energetically reasonable configurations near the system’s minimal energy configuration, while also obeying the biological mechanisms described within the terms in the effective energy equation.

### Cell volume and surface

Each cell has a defined volume and surface area (Table A), which contribute to the effective energy equation as:

|  | $H= \sum_{\sigma} {\lambda_{vol}\left( \sigma\right)\left( v\left( \sigma\right)-V_{t}\left( \sigma\right) \right)}^{2}+ \sum_{\sigma} {\lambda_{surf}\left( \sigma\right)\left( s\left( \sigma\right)- S_{t}\left( \sigma\right) \right)}^{2}$ | 3 |
| --- | --- | --- |

Where $v(\sigma)$ and s$(\sigma)$ represent a cell’s instantaneous volume and surface, and $V_{t}\left( \sigma\right)$ and $S_{t}\left( \sigma\right)$ represent the target volume and target surface, respectively. In addition, $\lambda_{vol}\left( \sigma\right)$ and $\lambda_{surf}\left( \sigma\right)$ represent the volume and surface constraints of the cell and can be correlated to Young’s modulus (i.e., modulus of elasticity). The λ values used in the model are provided in Table A and are assumed to be the same for every cell type, similar to previous work by [1].

### Cell adhesion

Adhesion and repulsion between neighbouring cells are represented in the effective energy equation by the function:

|  | $H= \sum_{i,j} J(\tau(\sigma_{i}),\tau(\sigma_{j})))(1-\delta(\sigma_{i},\sigma_{j}))$ | 4 |
| --- | --- | --- |

Where $\sigma_{i},\sigma_{j}$ are the IDs of the cells to which pixels *i* and *j* belong, $\tau(\sigma$*)* is the type of cell *σ*, $J$ is the contact energy between cells at *i* and *j*, and $\delta$ is the Kroenecker delta function given as,

|  | $\delta\left( \sigma_{i},\sigma_{j} \right)=\left\{ \begin{aligned} 0, &\sigma_{i}\neq\sigma_{j} \\ 1, &\sigma_{i}=\sigma_{j} \end{aligned} \right.$ | 5 |
| --- | --- | --- |

The term $(1-\delta\left( \sigma_{i},\sigma_{j} \right))$ will ensure that only pixels belonging to different cells will be considered in the sum of contact energies between neighbouring pixels [2]. The contact energies between different cell types are given in Table A. Generally, a higher contact energy results in repulsion, whereas a lower contact energy leads to adhesion between cells.

### Chemotaxis

Fibroblast and myofibroblast chemotaxis towards PDGF [3] is represented in the effective energy equation as,

|  | $\Delta E_{PDGF} = {-\lambda}_{PDGF}(\left[ PDGF \right]\left( \vec{x} \right)- \left[ PDGF \right]\left( \vec{x}^{'} \right))$ | 6 |
| --- | --- | --- |

Where $\lambda_{PDGF}$ is the chemotaxis strength coefficient, $\left[ PDGF \right]\left( \vec{x} \right)$ is the concentration of PDGF at the source site and $\left[ PDGF \right]\left( \vec{x}^{'} \right)$ is the concentration at the target site.

Similarly, macrophage chemotaxis towards the inflammation field produced by senescent cells is represented as [4],

|  | $\Delta E_{INF} = {-\lambda}_{INF}(\left[ INF \right]\left( \vec{x} \right)- \left[ INF \right]\left( \vec{x}^{'} \right))$ | 7 |
| --- | --- | --- |

### Model parameter estimation

One of the most important constructs when modelling are the parameters – values which alter the model behaviour as presented in the equations describing model mechanics, as well as the various threshold parameters described thus far. Model parameters were obtained from literature where possible. However, direct experimental measurements or accurate estimates were not always available. Therefore, where experimental evidence was available, parameter values were obtained from qualitative sources from literature. Where this was not possible, parameter values were simulated from theoretically sound estimates to produce a biologically reasonable representation of the tissue repair process based on the information gathered from literature. For parameters for which even an estimate is challenging, Particle Swarm Optimisation (PSO) was used by fitting the model behaviour to data gathered from literature shown in Fig A for fibroblasts, macrophages, myofibroblasts, senescent myofibroblasts and the rate of wound closure. PSO is an algorithm commonly used in computational modelling to identify likely values for parameters by using populations of candidate solutions, called swarms, and a measure of the quality of candidate solutions[5][6]. Briefly, PSO treats a particular set of parameters as a point (particle) in a multidimensional parameter space. For each particle, a quality metric is calculated using the model. The PSO generate multiple such parameter particles that propagate through parameter space looking for an optimal solution. Individual particles change their values based on the best result they have encountered as well as the best point encountered by the entire swarm. A swarm of particles will coalesce on a particular set of parameters in the parameter space, which may or may not be a global minimum. Multiple independent swarms are used to ensure adequate sampling of the entire parameter space. If multiple swarms find the same set of parameters, then that suggests the solution is either a global minimum, or at least a local minimum that is likely to be similar in quality to the (unknown) global minima.

The total number of swarms was 2, with 16 particles each and 2 duplicate runs (since CC3D simulations are stochastic) which were run for 60 iterations. Model parameter ranges for optimisation were determined by running the initial model several times with various parameter sets. The final set of ranges is summarised in Table B. Parameter estimation using PSO for this model was run on a Linux cluster hosted by Indiana University.

#### Data used for PSO

Quality of fit for candidate solutions was determined using relative error between simulation output and data from literature where possible. This includes cell population profiles for fibroblasts, myofibroblasts, macrophages and senescent myofibroblasts, ECM production and rate of wound closure, which are shown in Fig A.

Fibroblast and macrophage curves were informed using a study by Zhou et al., where an *in vitro* system of murine fibroblasts and macrophages, interacting via growth factor exchange (PDGF for fibroblasts and CSF1 for macrophages), was used to investigate the stability of the two-cell circuit [7]. The results from this study, which showed that fibroblasts and macrophages form a stable cell circuit, was also used in another study by Adler et al. to investigate the myofibroblast and macrophage cell circuit in tissue repair and fibrosis [8]. Hence, this data was used, along with the curves depicting the timeline of cellular activity from Witte et al., to inform the fibroblast and macrophage curves [9].

The curve for senescent myofibroblasts was informed using the data from Jun et al., where a mouse model of cutaneous wound healing was used to investigate CCN1-induced senescence in wound healing and fibrosis [10]. The study reported that senescent cells, which constituted 15% of all cells, accumulated in wounds between 7-9 days post-wounding; these senescent cells were identified as myofibroblasts through biomarkers. The curve for myofibroblasts was obtained from McAndrews et al. where a murine wound healing model was used to investigate myofibroblast function in wound healing [11].

The rate of wound closure curve for human wounds was obtained from Bain et al. [12]. Acquiring ECM data was particularly challenging since most studies investigate particular types of collagen or other components of the ECM, which have crucial functions in the wound healing process. For the sake of simplicity and computational feasibility, ECM in this model was considered as a single entity, instead of modelling each component explicitly. In this respect, the curve for ECM was informed by Adler et al. [8] and Urciuolo et al. [13]. The data was calibrated and converted to numerical data using the online tool WebPlotDigitizer [14].

Model parameter ranges for PSO were selected with the aim of minimising the time and iterations required by the PSO algorithm to converge to an appropriate parameter set with minimal error. With that in mind, multiple preliminary PSO runs (10 prior runs of 2 swarms, with 16 particles each with 2 replicates and 60 iterations) led to the identification of optimal approximate parameter ranges as shown in Table B.


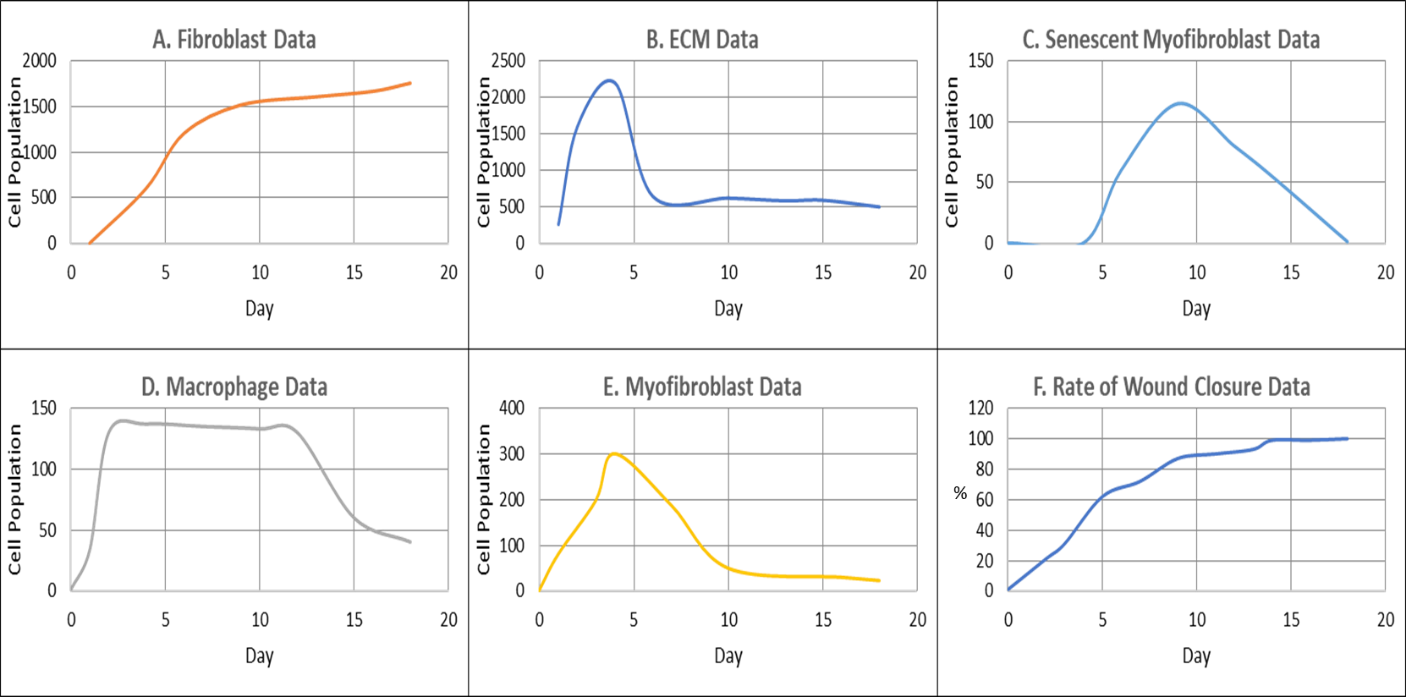


Fig A. **Data used for PSO.**

Curves show the timeline for cellular and ECM levels across the duration of wound healing, calibrated to the model. These curves were informed using existing studies, and are shown for **A:** Fibroblasts, **B:** ECM, **C:** Senescent myofibroblasts, **D:** Macrophages, **E:** Myofibroblasts, **F:** Rate of wound closure. Days are shown on the x axis and cell population and percentage of wound closure are shown on the y axis.

### Simulation specifications

A baseline parameter set was established for all CPM (CC3D) parameters and model mechanisms described in the above sections. These are provided in Tables A and B. Simulation data was collected at a frequency of 500 MCS (every 3.75 simulated hours) for each model run. Three replicate runs were performed.

## Supplementary results

### A multiscale model of healthy wound healing

To summarise, cell types within the model were represented by collections of pixels with unique identifiers. Cells interact with each other based on parameters that were obtained from literature or estimated. The parameters steer model behaviour through the effective energy or Hamiltonian equation, with the simulation evolving stochastically through a Monte Carlo with a Boltzmann acceptance function. Growth factors and cytokines within the model were defined using reaction diffusion PDEs. The wound healing model had a large number of parameters, some of which were obtained from literature, summarised in Table A, whereas others were estimated using PSO. The best parameter sets identified by PSO are given in Table B. Summary of the output from running PSO is in the Table C. PSO was run for a total of 60 iterations, and the best parameter set was found at the 46th iteration. Parameter values from swarm 1 were used for the final model as it had a slightly better fitness measure (relative error of 27.84). Despite the effort put into making the model parameters as physiologically and biologically relevant as possible, it was not possible to match or find appropriate data for some parameters. For these, parameter values were simulated from theoretically sound estimates to produce a biologically reasonable representation of the wound healing process based on information gathered from literature, as previously stated, and provided in Table A.

### Hamiltonian equation for a multiscale model of healthy wound healing including senescent cell dynamics

Parameters steer model behaviour through the Hamiltonian equation given as:

|  | $H= \sum_{i,j} J(\tau(\sigma_{i}),\tau(\sigma_{j})))(1-\delta(\sigma_{i},\sigma_{j}))+\sum_{\sigma} {\lambda_{vol}\left( \sigma\right)\left( v\left( \sigma\right)-V_{t}\left( \sigma\right) \right)}^{2}+ \sum_{\sigma} {\lambda_{surf}\left( \sigma\right)\left( s\left( \sigma\right)- S_{t}\left( \sigma\right) \right)}^{2}+ \Delta E_{PDGF}+ \Delta E_{INF}$ | 8 |
| --- | --- | --- |

Where each cell *σ* possesses a cell type $\tau(\sigma)$ and is represented by pixels *i* and *j*. The first term in the Hamiltonian equation represents intercellular adhesion, where $J$ is the contact energy between cells at *i* and *j*, and $\delta$ is the Kronecker delta function which ensures that only contact energies between pixels from different cells are included in the energy calculation. The second and third terms describe the volume and surface area of cells, where $v(\sigma)$ and s$(\sigma)$ are the number of pixels within a cell and comprising the cell perimeter, respectively. $V_{t}\left( \sigma\right)$ and $S_{t}\left( \sigma\right)$ are the target volume and target surface, respectively, whereas $\lambda_{vol}\left( \sigma\right)$ and $\lambda_{surf}\left( \sigma\right)$ are volume and surface constraints, respectively. The last two terms in the equation represent cell chemotaxis towards the PDGF and inflammation chemical fields. The simulation evolves stochastically through a Monte Carlo with a Boltzmann acceptance function. At each time step, the algorithm randomly selects a source site $\vec{i}$ and a target site $\vec{i} '$. The change in the effective energy $\Delta H$ required to copy the index from the source to the target site is then calculated, which in turn determines the probability of accepting a new model configuration.

## Supplementary figures


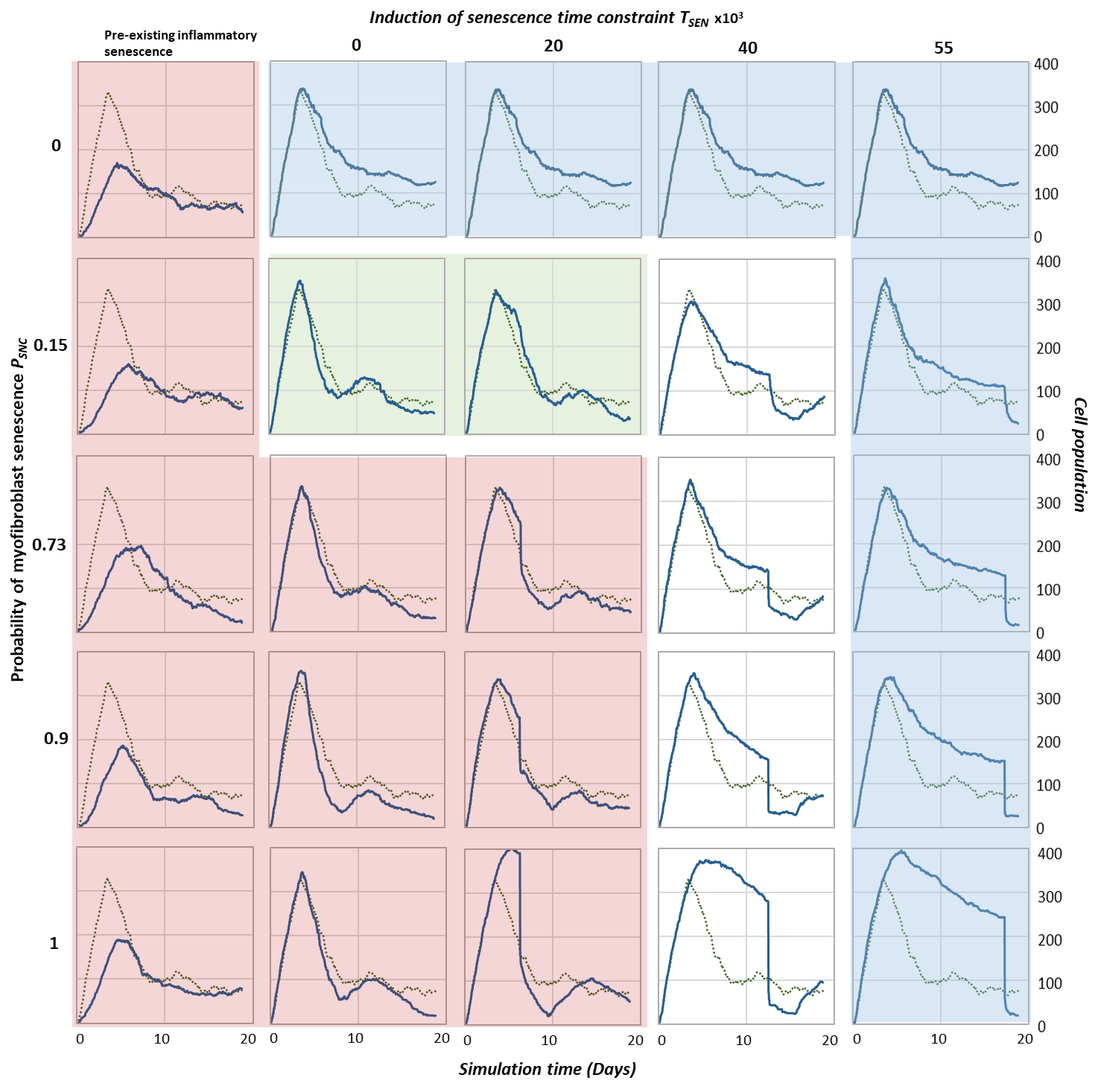


Fig B. **Wound healing model** **multidimensional sensitivity analysis of the number of myofibroblast vs time for variations in the parameters probability of myofibroblast senescence (*P_SNC_*) and senescence induction time constraint (*T_SEN_*) showing regions with distinct repair dynamics.**

The subplots show the model simulation time series for myofibroblasts in the wound from a pairwise parameter sweep of the parameters: probability of myofibroblast senescence *P_SNC_* (shown vertically on the left) and senescence induction time constraint *T_SEN_* (shown horizontally at the top), around their baseline values (provided in Table A). Along with the different values included in the parameter sweep for the senescence induction time constraint *T_SEN_* parameter, pre-existing inflammatory senescent cells are also shown to represent senescence induced during the inflammatory phase of the wound healing process (i.e., before *T_SEN_* = 0) which was not explicitly included in the model. Simulation time in days is shown on the x axis and cell population numbers are shown on the y axis. The boxes highlight regions with distinct repair dynamics: chronic wound inflammation (red shaded box), healthy healing (green shaded box) and fibrotic wound response (blue shaded box). The solid blue line represents simulation time series from the pairwise parameter sweep. The dotted lines in all the plots represent simulation time series from the healthy physiological wound healing model included for comparison.


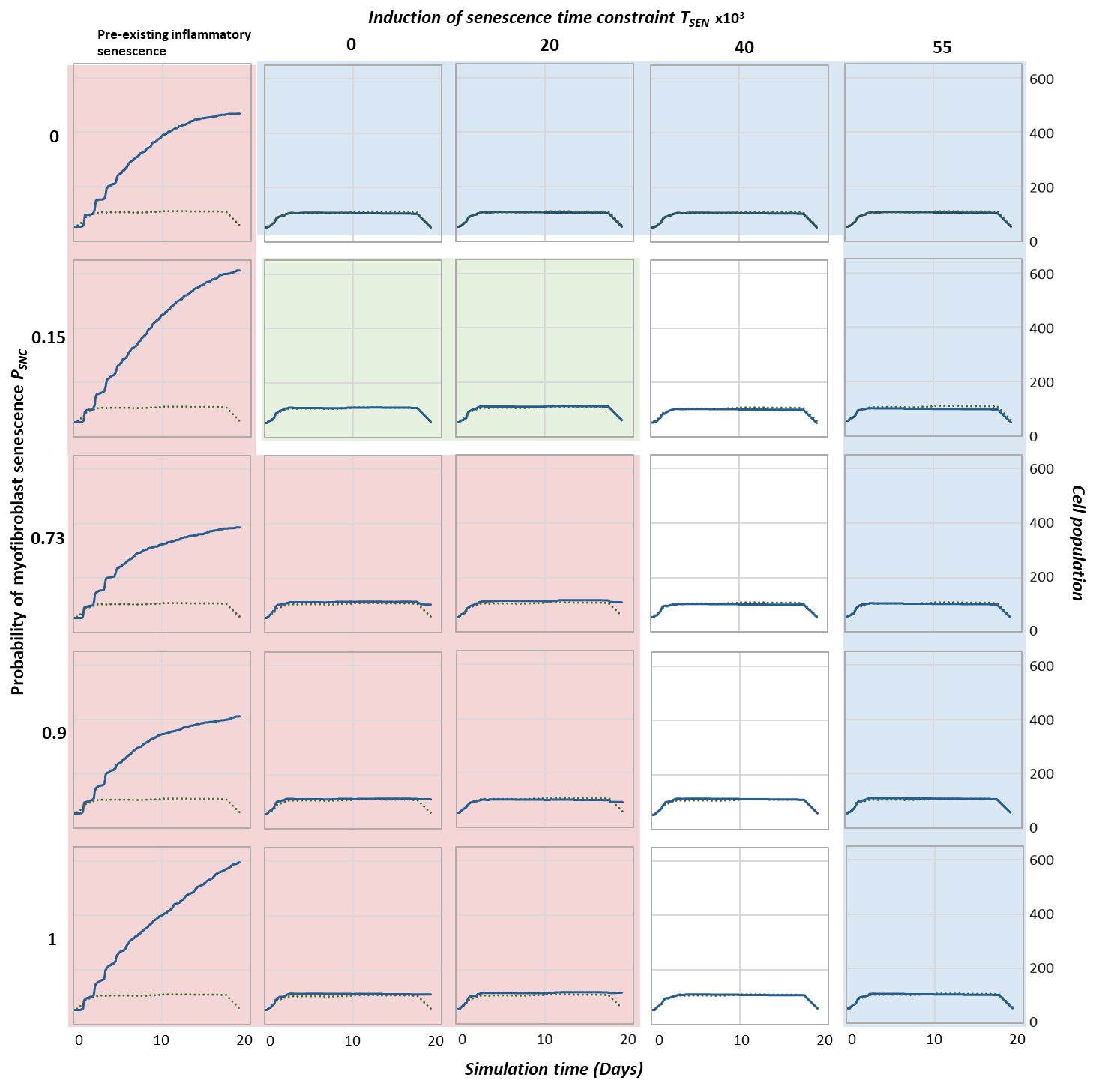


Fig C. **Wound healing model** **multidimensional sensitivity analysis of the number of macrophages vs time for variations in the parameters probability of myofibroblast senescence (*P_SNC_*) and senescence induction time constraint (*T_SEN_*) showing regions with distinct repair dynamics.**

The subplots show the model simulation time series for macrophages in the wound from a pairwise parameter sweep of the parameters: probability of myofibroblast senescence *P_SNC_* (shown vertically on the left) and senescence induction time constraint *T_SEN_* (shown horizontally at the top), around their baseline values (provided in Table A). Along with the different values included in the parameter sweep for the senescence induction time constraint *T_SEN_* parameter, pre-existing inflammatory senescent cells are also shown to represent senescence induced during the inflammatory phase of the wound healing process (i.e., before *T_SEN_* = 0) which was not explicitly included in the model. Simulation time in days is shown on the x axis and cell population numbers are shown on the y axis. The boxes highlight regions with distinct repair dynamics: chronic wound inflammation (red shaded box), healthy healing (green shaded box) and fibrotic wound response (blue shaded box). The solid blue line represents simulation time series from the pairwise parameter sweep. The dotted lines in all the plots represent simulation time series from the healthy physiological wound healing model, included for comparison.


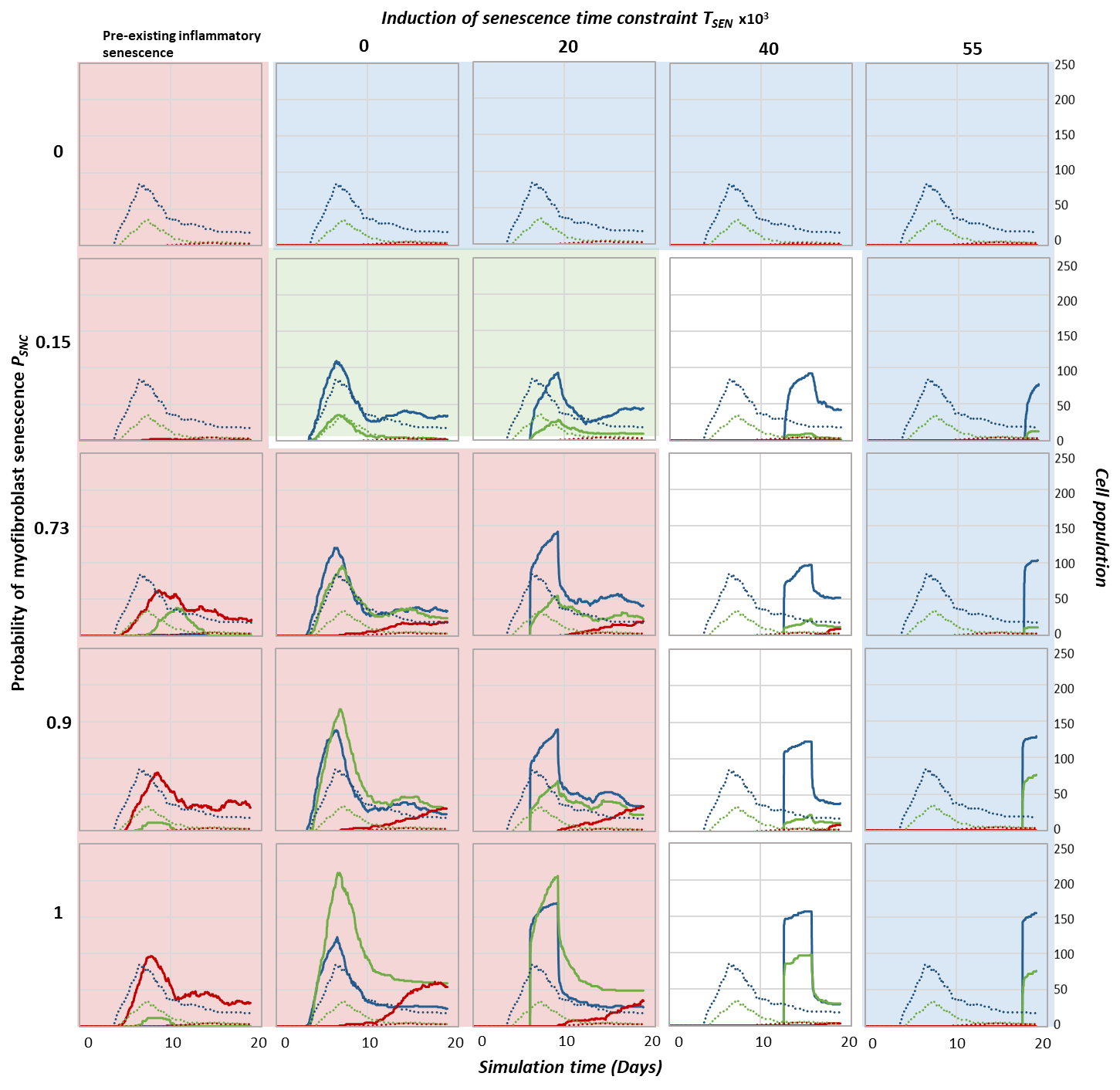


Fig D. **Wound healing model** **multidimensional sensitivity analysis of the number of senescent cells including senescence induction mechanism vs time for variations in the parameters probability of myofibroblast senescence (*P_SNC_*) and senescence induction time constraint (*T_SEN_*) showing regions with distinct repair dynamics.**

The subplots show the model simulation time series for senescent cell numbers in the wound from a pairwise parameter sweep of the parameters: probability of myofibroblast senescence *P_SNC_* (shown vertically on the left) and senescence induction time constraint *T_SEN_* (shown horizontally at the top), around their baseline values (provided in Table A). Along with the different values included in the parameter sweep for the senescence induction time constraint *T_SEN_* parameter, pre-existing inflammatory senescent cells are also shown to represent senescence induced during the inflammatory phase of the wound healing process (i.e., before *T_SEN_* = 0) which was not explicitly included in the model. Simulation time in days is shown on the x axis and cell population numbers are shown on the y axis. The boxes highlight regions with distinct repair dynamics: chronic wound inflammation (red shaded box), healthy healing (green shaded box) and fibrotic wound response (blue shaded box). The plot colours represent different modes of senescence induction; red: paracrine secondary senescence, blue: juxtacrine secondary senescence and green: CCN1-induced primary senescence. The solid lines represent simulation time series from the pairwise parameter sweep. The dotted lines represent simulation time series from the healthy physiological wound healing model were included for comparison.


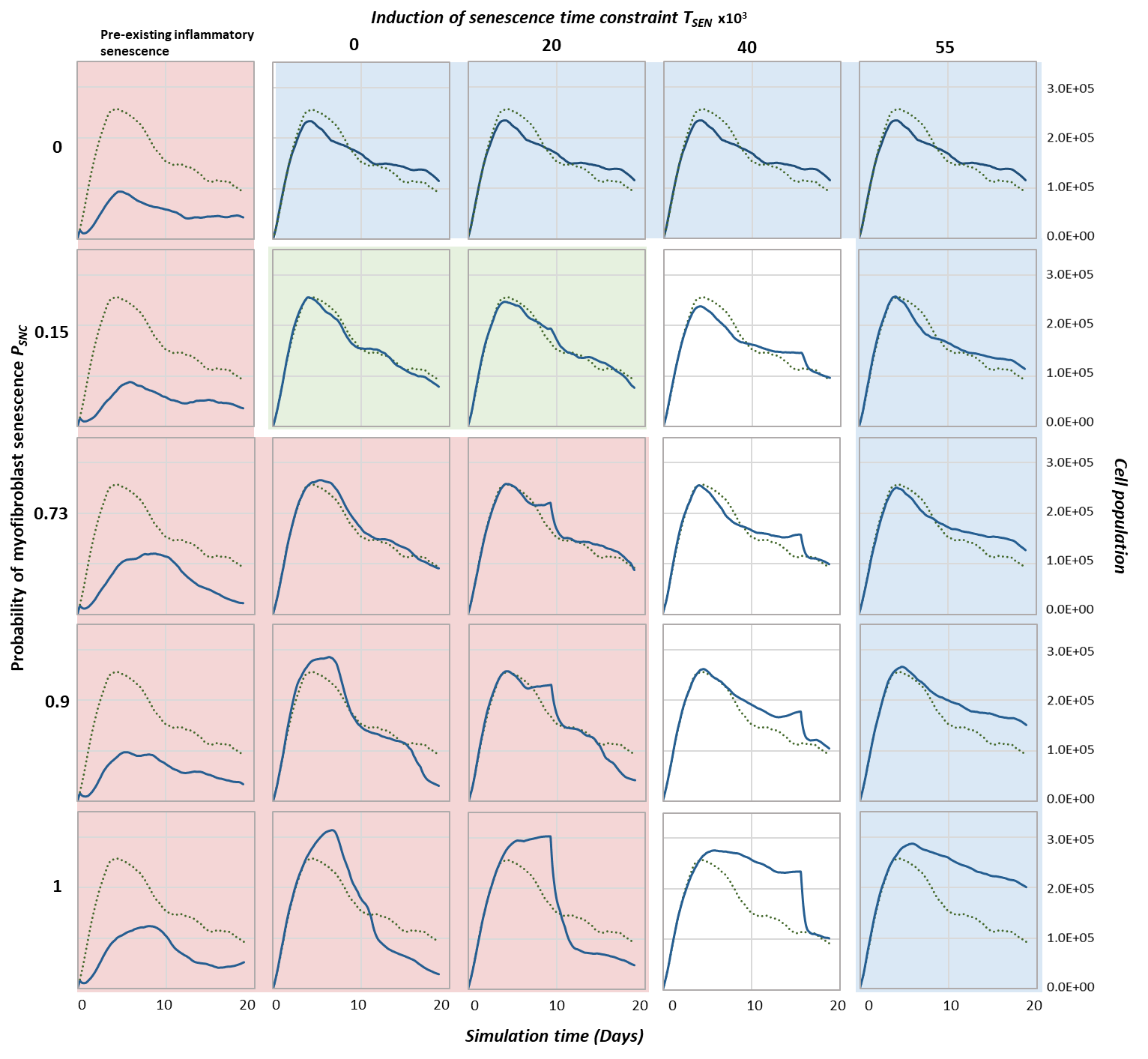


Fig E. **Wound healing model** **multidimensional sensitivity analysis of total PDGF vs time for variations in the parameters probability of myofibroblast senescence (*P_SNC_*) and senescence induction time constraint (*T_SEN_*) showing regions with distinct repair dynamics.**

The subplots show the model simulation time series for PDGF levels in the wound from a pairwise parameter sweep of the parameters: probability of myofibroblast senescence *P_SNC_* (shown vertically on the left) and senescence induction time constraint *T_SEN_* (shown horizontally at the top), around their baseline values (provided in Table A). Along with the different values included in the parameter sweep for the senescence induction time constraint *T_SEN_* parameter, pre-existing inflammatory senescent cells are also shown to represent senescence induced during the inflammatory phase of the wound healing process (i.e., before *T_SEN_* = 0) which was not explicitly included in the model. Simulation time in days is shown on the x axis and chemical concentration levels are shown on the y axis. The boxes highlight regions with distinct repair dynamics: chronic wound inflammation (red shaded box), healthy healing (green shaded box) and fibrotic wound response (blue shaded box). The solid blue line represents simulation time series from the pairwise parameter sweep. The dotted lines in all the plots represent simulation time series from the healthy physiological wound healing model were included for comparison.


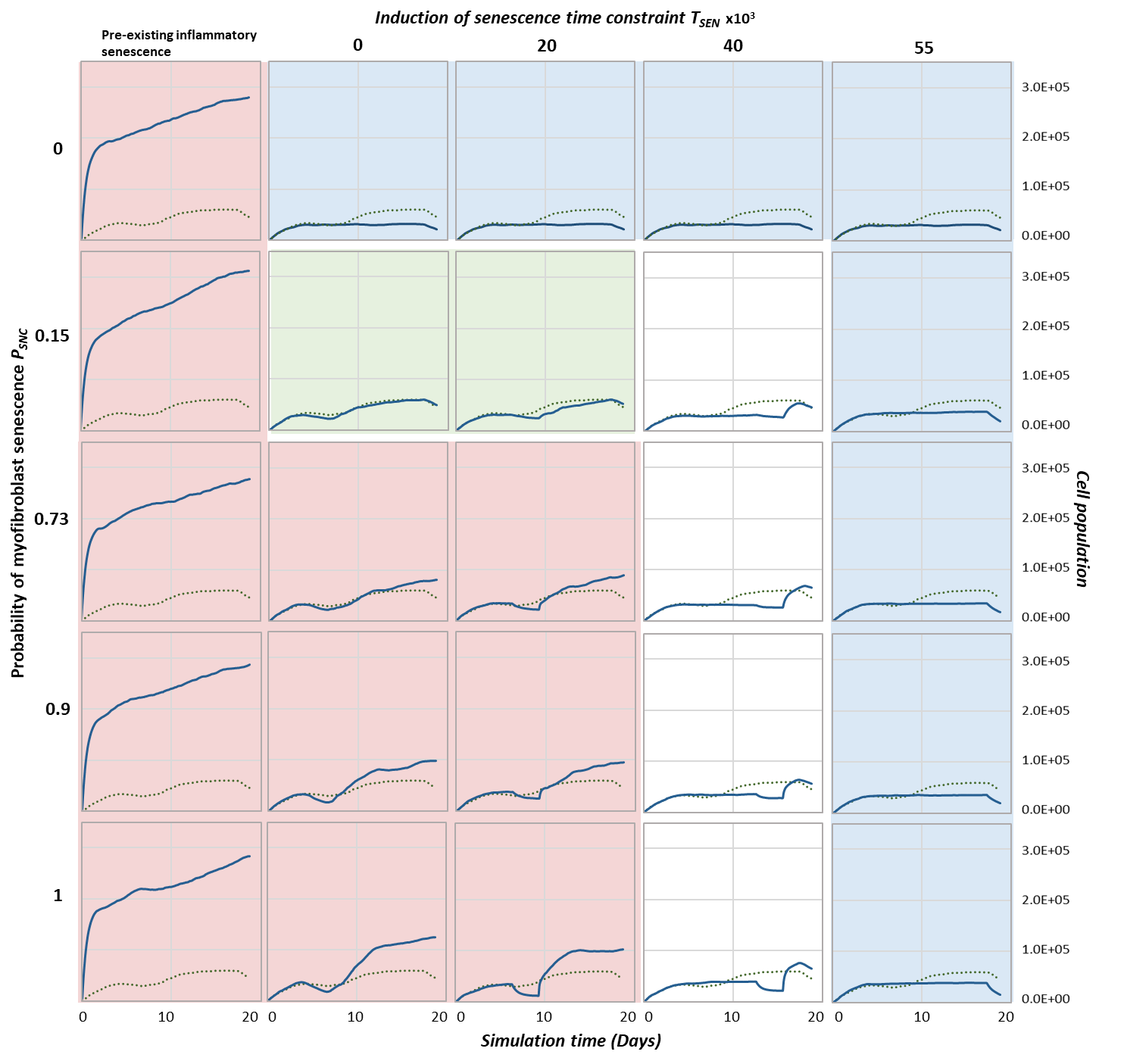


Fig F. **Wound healing model** **multidimensional sensitivity analysis of total CSF1 vs time for variations in the parameters probability of myofibroblast senescence (*P_SNC_*) and senescence induction time constraint (*T_SEN_*) showing regions with distinct repair dynamics.**

The subplots show the model simulation time series for CSF1 levels in the wound from a pairwise parameter sweep of the parameters: probability of myofibroblast senescence *P_SNC_* (shown vertically on the left) and senescence induction time constraint *T_SEN_* (shown horizontally at the top), around their baseline values (provided in Table A). Along with the different values included in the parameter sweep for the senescence induction time constraint *T_SEN_* parameter, pre-existing inflammatory senescent cells are also shown to represent senescence induced during the inflammatory phase of the wound healing process (i.e., before *T_SEN_* = 0) which was not explicitly included in the model. Simulation time in days is shown on the x axis and chemical concentration levels are shown on the y axis. The boxes highlight regions with distinct repair dynamics: chronic wound inflammation (red shaded box), healthy healing (green shaded box) and fibrotic wound response (blue shaded box). The solid blue line represents simulation time series from the pairwise parameter sweep. The dotted lines in all the plots represent simulation time series from the healthy physiological wound healing model were included for comparison.


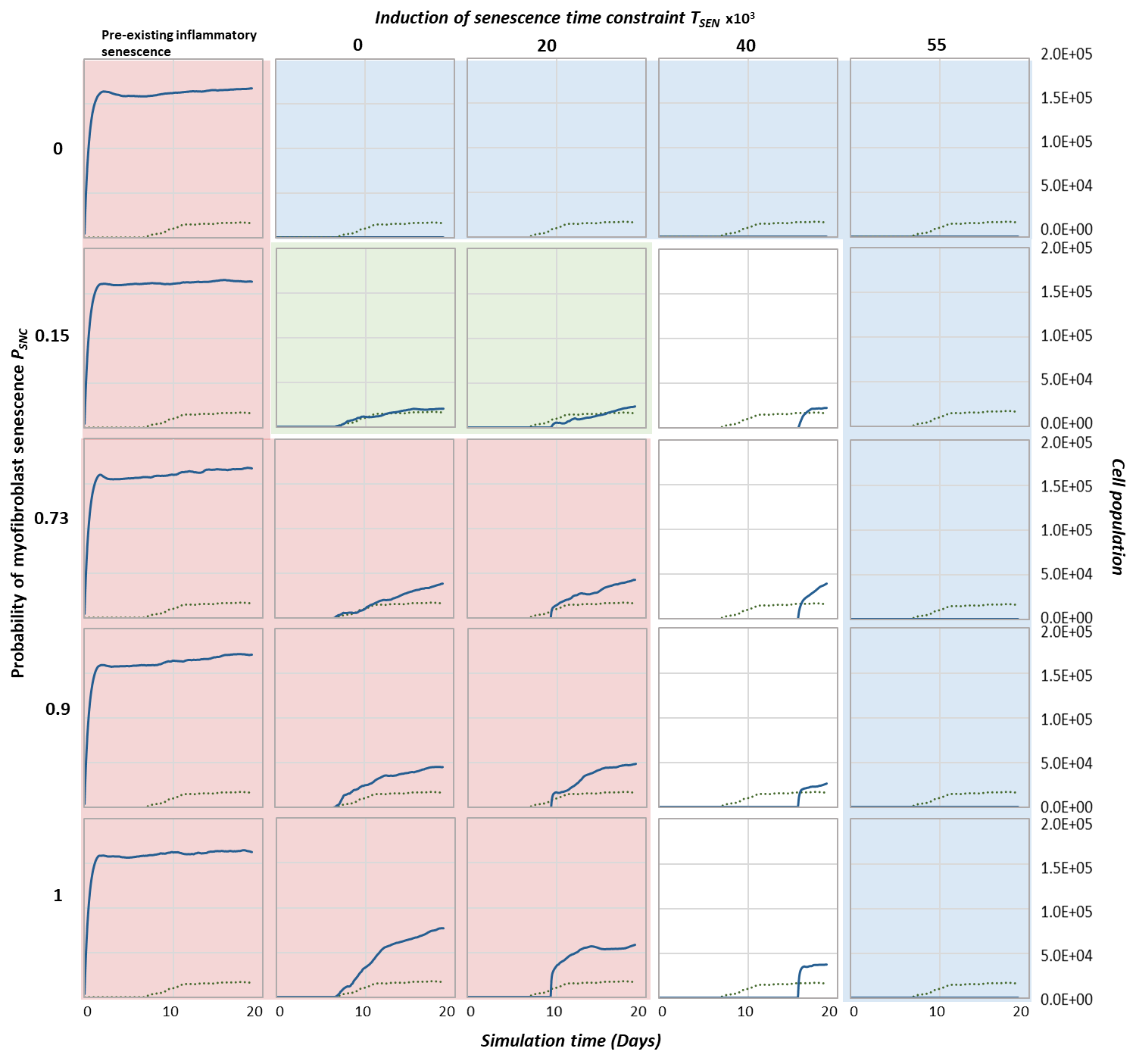


Fig G. **Wound healing model** **multidimensional sensitivity analysis of total inflammatory SASP vs time for variations in the parameters probability of myofibroblast senescence (*P_SNC_*) and senescence induction time constraint (*T_SEN_*) showing regions with distinct repair dynamics.**

The subplots show the model simulation time series for inflammatory SASP levels in the wound from a pairwise parameter sweep of the parameters: probability of myofibroblast senescence *P_SNC_* (shown vertically on the left) and senescence induction time constraint *T_SEN_* (shown horizontally at the top), around their baseline values (provided in Table A). Along with the different values included in the parameter sweep for the senescence induction time constraint *T_SEN_* parameter, pre-existing inflammatory senescent cells are also shown to represent senescence induced during the inflammatory phase of the wound healing process (i.e., before *T_SEN_* = 0) which was not explicitly included in the model. Simulation time in days is shown on the x axis and chemical concentration levels are shown on the y axis. The boxes highlight regions with distinct repair dynamics: chronic wound inflammation (red shaded box), healthy healing (green shaded box) and fibrotic wound response (blue shaded box). The solid blue line represents simulation time series from the pairwise parameter sweep. The dotted lines in all the plots represent simulation time series from the healthy physiological wound healing model were included for comparison.


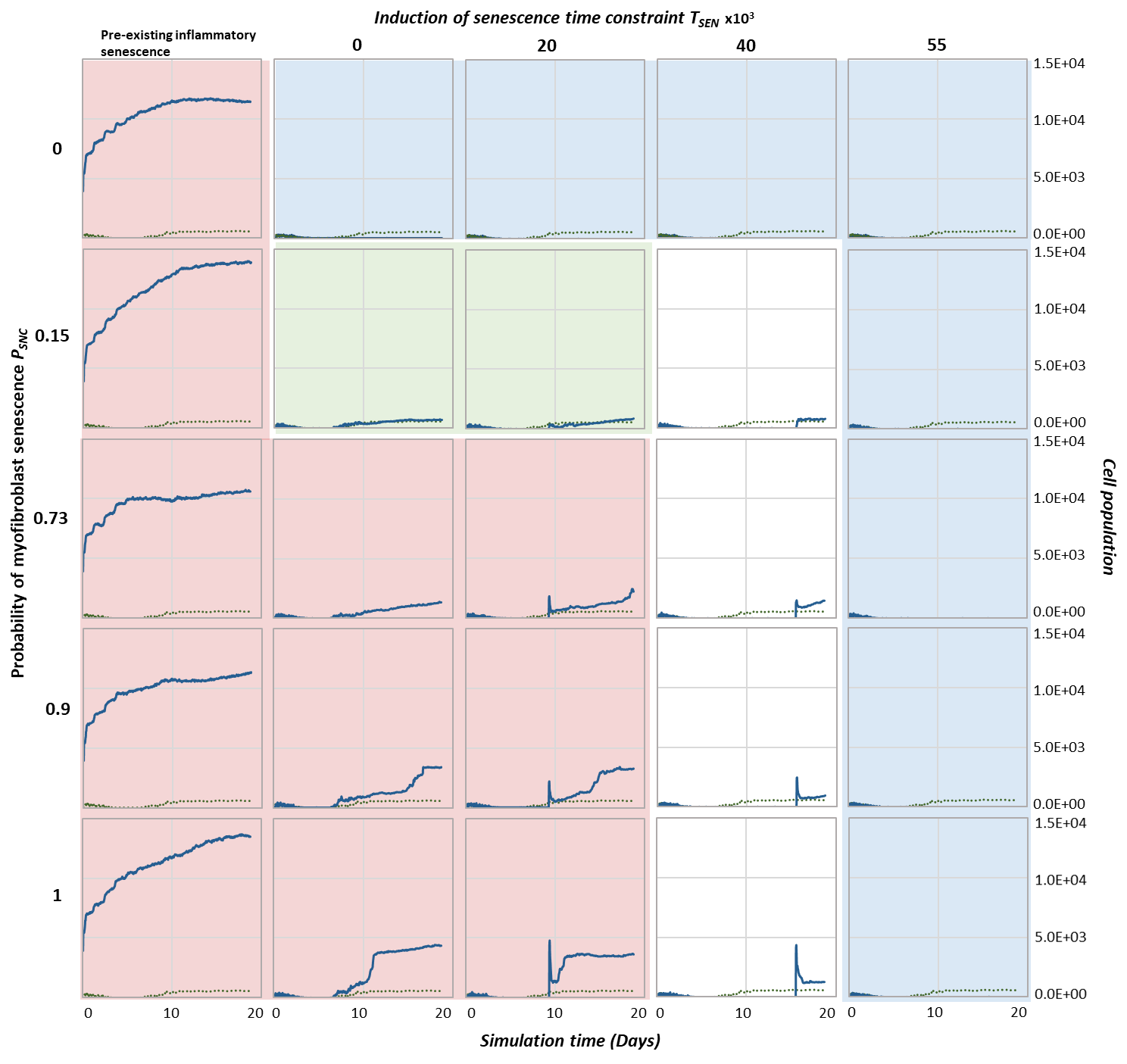


Fig H. **Wound healing model** **multidimensional sensitivity analysis of total MMP vs time for variations in the parameters probability of myofibroblast senescence (*P_SNC_*) and senescence induction time constraint (*T_SEN_*) showing regions with distinct repair dynamics.**

The subplots show the model simulation time series for MMP levels in the wound from a pairwise parameter sweep of the parameters: probability of myofibroblast senescence *P_SNC_* (shown vertically on the left) and senescence induction time constraint *T_SEN_* (shown horizontally at the top), around their baseline values (provided in Table A). Along with the different values included in the parameter sweep for the senescence induction time constraint *T_SEN_* parameter, pre-existing inflammatory senescent cells are also shown to represent senescence induced during the inflammatory phase of the wound healing process (i.e., before *T_SEN_* = 0) which was not explicitly included in the model. Simulation time in days is shown on the x axis and chemical concentration levels are shown on the y axis. The boxes highlight regions with distinct repair dynamics: chronic wound inflammation (red shaded box), healthy healing (green shaded box) and fibrotic wound response (blue shaded box). The solid blue line represents simulation time series from the pairwise parameter sweep. The dotted lines in all the plots represent simulation time series from the healthy physiological wound healing model were included for comparison.

## Supplementary tables

Table A. **Baseline parameter values and conversion factors for the wound healing model.**

| **Conversion Factors** | | **Value** | | **Reference/Rationale** | | | | |
| --- | --- | --- | --- | --- | --- | --- | --- | --- |
| Simulation time step Δt (Monte Carlo step, MCS) | | 27 seconds | | Cells in the simulation have an average speed of ~0.1 pixel/MCS (0.2 microns/MCS). This was determined by running multiple simulations of a cell without any hindrance and taking an average of the difference between the current and previous COM values [15]. As a reference, fibroblast migration speed during wound healing was considered, which peaks towards the end of the healing process with a speed of 40 µm/h [16]. Therefore, 1 MCS was calculated to be 27 secs (0.3x3600/40). | | | | |
| 1 voxel or pixel width | | 3 µm | | Selected based on diameters of cells. | | | | |
| Simulation lattice size | | 200 x 200 pixels | | Varied and selected based on the appropriate size of the wound and computational feasibility. | | | | |
| **Simulation Parameters** | | **Value** | | **Reference/Rationale** | | | | |
| Cell membrane fluctuation amplitude $T_{m}$ | | 30 | | Estimated based on generating significant cell motility given the time equivalent of one MCS. | | | | |
| Periodic Boundary Conditions: Cells | | X-axis: no  Y-axis: no | | Cell behaviour at the limits of the simulated region. Cells wrap around at periodic boundaries and do not at non-periodic boundaries. | | | | |
| Periodic Boundary Conditions: Diffusing Fields | | X-axis: no  Y-axis: no | | Field behaviour at the limits of the simulated region. Fields wrap around at periodic boundaries and do not at non-periodic boundaries. | | | | |
| PDGF diffusion coefficient $D_{PDGF}$ | | 0.01mm^2^/h | | Value selected was for diffusion through normal tissue [17], but diffusion could be slower in dense ECM [18]. Therefore, diffusion coefficient was taken to be 50% slower. | | | | |
| PDGF decay constant $\delta_{PDGF}$ | | 2/day | | [8] | | | | |
| MMP diffusion coefficient $D_{CSF}$ | | 0.6x10^8^cm^2^/s | | Diffusion coefficient for MMP 9 on collagen fibrils [19] | | | | |
| MMP decay constant $\delta_{MMP}$ | | 2x10^-3^/s | | [20], [21] | | | | |
| Inflammatory cytokine diffusion coefficient $D_{INF}$ | | 0.01mm^2^/h | | Used the same value as the diffusion coefficient of PDGF since some inflammatory interleukins and PDGF have similar molecular weights [18, 22] | | | | |
| Inflammatory cytokine decay constant $\delta_{INF}$ | | 2/day | | Used the same value as the decay constant of PDGF since some inflammatory interleukins and PDGF have similar molecular weights | | | | |
| CSF1 diffusion coefficient $D_{CSF}$ | | 3.33x10^-3^ mm^2^/h | | Molecular weight of CSF1 is 60 kDa, therefore diffusion coefficient was assumed to be 1/3^rd^ that of PDGF [18, 22] | | | | |
| CSF1 decay constant $\delta_{CSF}$ | | 2/day | | [8] | | | | |
| PDGF secretion rate $S_{PDGF}$ | | 1 | | Estimated | | | | |
| MMP secretion rate $S_{MMP}$ | | 1 | | Estimated | | | | |
| Inflammatory cytokine secretion rate $S_{INF}$ | | 1 | | Estimated | | | | |
| CSF1 secretion rate $S_{CSF}$ | | 1 | | Estimated | | | | |
| PDGF molecular weight | | 30 kDa | | [23] | | | | |
| CSF1 molecular weight | | 60 kDa | | [24] | | | | |
| Inflammatory cytokine molecular weight | | 23 kDa | | Estimated to be the molecular weight of interleukins [24] | | | | |
| MMP molecular weight | | 80 kDa | | Estimated to be the molecular weight of MMP9 [24] | | | | |
| Cell spatial characteristics | | | | | | | | |
| Fibroblast target volume | | 196 µm^2^ | | Unit for 2D simulation [25] | | | | |
| Fibroblast target surface | | 56 µm | | Unit for 2D simulation [25] | | | | |
| Macrophage target volume | | 324 µm^2^ | | Unit for 2D simulation (BNID103565) | | | | |
| Macrophage target surface | | 72 µm | | Unit for 2D simulation (BNID103565) | | | | |
| Senescent fibroblast target volume | | 196 µm^2^ | | Unit for 2D simulation (BNID108244) | | | | |
| Senescent fibroblast target surface | | 56 µm | | Unit for 2D simulation (BNID108244) | | | | |
| Myofibroblast target volume | | 324 µm^2^ | | Unit for 2D simulation. (Estimate) | | | | |
| Myofibroblast target surface | | 72 µm | | Unit for 2D simulation. (Estimate) | | | | |
| Lambda volume $\lambda_{vol}$ | | 2 | | Same lambda volume value was used for all cells for appropriate cell volume deformation (Estimate) | | | | |
| Lambda surface $\lambda_{surf}$ | | 2 | | Same lambda surface value was used for all cells for appropriate cell surface deformation (Estimate) | | | | |
| Cell transition parameters | | | | | | | | |
| Probability of myofibroblast senescence *P_SNC_* | | 0.15 | | Previous work has shown that the percentage of senescent myofibroblasts in healthy wound healing was ~15-18% [10] | | | | |
| Probability of myofibroblast differentiation  *P_MF_* | | 0.2 | | Percentage of myofibroblast cells in wound healing has previously been shown to be approximately ~20-25% [26] | | | | |
| PDGF concentration at half maximum fibroblast growth ${PDGF}_{0}$ | | 49.8 pg/ml | | [23] | | | | |
| CSF1 concentration at half maximum macrophage growth ${CSF}_{0}$ | | 141.15 pg/ml | | [8] | | | | |
| Fibroblast max growth rate $G_{\max(F,MF)}$ | | 0.9/day | | [8] | | | | |
| Macrophage max growth rate $G_{max(M)}$ | | 0.8/day | | [8] | | | | |
| Fibroblast activation PDGF threshold *PDGF_f_* | | 0.5 | | Estimated | | | | |
| Myofibroblast juxtacrine senescence PDGF threshold *SNC_thr_* | | Varied. | | Estimated using PSO | | | | |
| Proteinase threshold for ECM breakdown *MMP_thr_* | | Varied | | Estimated using PSO | | | | |
| Myofibroblast deactivation ECM threshold *ECM_thr_* | | Varied | | Estimated using PSO | | | | |
| Inflammatory SASP threshold *INF_thr_* | | Varied | | Estimated using PSO | | | | |
| CSF1 threshold for macrophage depletion *CSF1_thr_* | | Varied | | Estimated using PSO | | | | |
| Contact inhibition threshold $T_{CI}$ | | 0.05 | | [27] | | | | |
| Base macrophage and fibroblast removal rate (apart from cell death due to growth factor depletion) μ | | 1 cell/3.3 days | | [8] | | | | |
| Probability of senescent cell clearance through immune surveillance by macrophages; removal by other immune cells not explicitly modelled | | 50%; 50% | | Estimated | | | | |
| Duration of notch induced senescence *T_NIS_* | | 3 days | | Preliminary work suggests that NOTCH-mediated juxtacrine secondary senescence induction and fibrogenic SASP are prevalent up to approximately 3 days [28, 29] | | | | |
| Senescence induction time constraint *T_SEN_* | | 12 days | | Estimated | | | | |
| Rate of ECM production by myofibroblasts *ECM_Myof_* | | 2 cells | | Selected to be most appropriate after running multiple preliminary model simulations with different parameter values. | | | | |
| Rate of ECM production by fibroblasts *ECM_Fib_* | | 1 cell | | Selected to be most appropriate after running multiple preliminary model simulations with different parameter values. | | | | |
| Probability of Myofibroblast de-differentiation; myofibroblast apoptosis | | 50%; 50% | | Approximated from [30] | | | | |
| Adhesion energies $J$  (note that CPM attempts to minimize energy, so smaller J values are more adhesive) | | | | | | | | |
| Cell Type | Medium | | Fibroblast | | Myofibroblast | Macrophage | Senescent fibroblast | ECM |
| Medium | 0 | | 1 | | -1 | -2 | -1 | -1 |
| Fibroblast | -- | | 2 | | 1 | -1 | 2 | 1 |
| Myofibroblast | -- | | -- | | -1 | -1 | -1 | 1 |
| Macrophage | -- | | -- | | -- | 2 | -1 | 1 |
| Senescent fibroblast | -- | | -- | | -- | -- | 5 | 1 |
| ECM | -- | | -- | | -- | -- | -- | -2 |

Table B. **Summary of PSO output for individual swarms.**

PSO was run with 2 swarms, 16 particles each, for 60 iterations. The best particle (i.e., parameter set) was identified at iteration 46, and is shown in the table. Swarm 1, which has a slightly lower relative error, was chosen to be used with the baseline parameter set.

| **Parameter** | **Swarm 1** | **Swarm 2** | **Relative standard deviation (%)** | **Min** | **Max** |
| --- | --- | --- | --- | --- | --- |
| Fibroblast activation PDGF threshold (*PDGF_f_)* | 8.1044e-02 | 5.7022e-02 | 17.4% | 0.05 | 2.0 |
| PDGF concentration threshold for myofibroblast juxtacrine senescence (*SNC_thr_*) | 4.2876e+00 | 4.7675e+00 | 5.3% | 1.0 | 5.0 |
| Proteinase threshold for ECM breakdown (*MMP_thr_*) | 2.0926e-02 | 2.1085e-02 | 0.4% | 0.02 | 2.0 |
| Myofibroblast deactivation ECM threshold (*ECM_thr_*) | 8.0205e+00 | 8.7545e+00 | 4.4% | 5.0 | 11.0 |
| Inflammation threshold (*INF_thr_*) | 2.3734e+00 | 2.8003e+00 | 8.3% | 1.0 | 3.0 |
| CSF1 threshold for macrophage depletion (*CSF1_thr_*) | 3.2847e+00 | 3.3808e+00 | 1.4% | 2.0 | 5.0 |
| Relative error value | 27.84 | 27.92 | -- | -- | -- |

Table C. **Summary of results from PSO**. The best swarm results are highlighted.


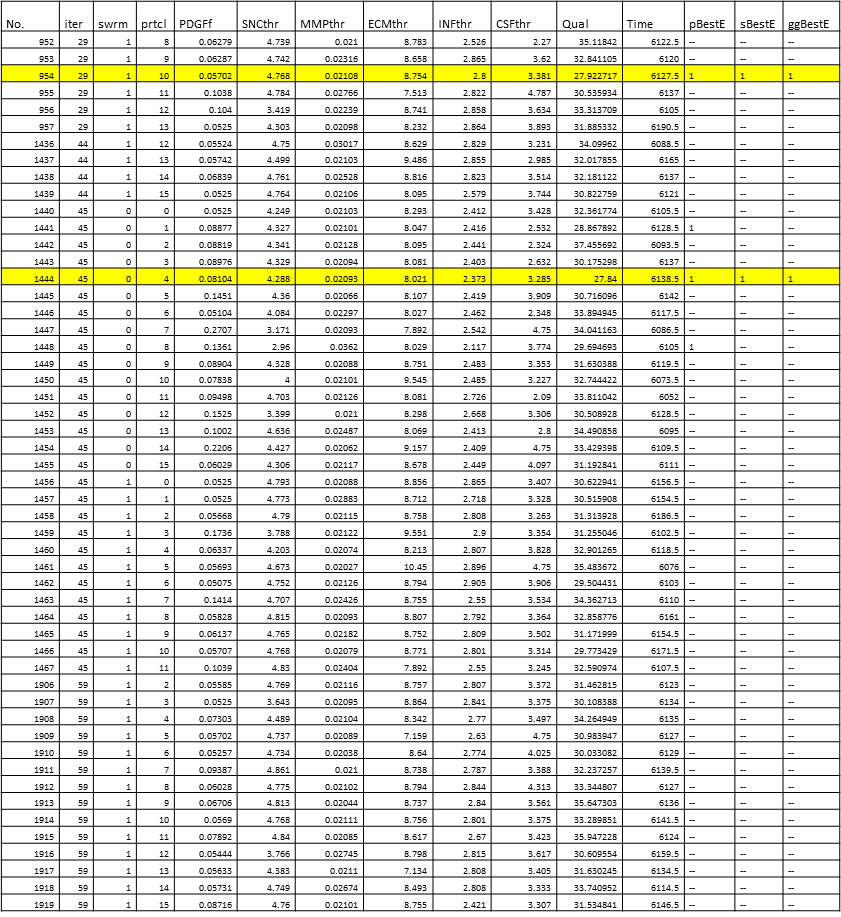


## References

1. Swat MH, Thomas GL, Shirinifard A, Clendenon SG, Glazier JA. Emergent stratification in solid tumors selects for reduced cohesion of tumor cells: A multi-cell, virtual-tissue model of tumor evolution using CompuCell3D. *PLoS One* 2015; 10: e0127972.

2. Swat MH, Thomas GL, Belmonte JM, Shirinifard A, Hmeljak D, Glazier JA. Multi-Scale Modeling of Tissues Using CompuCell3D. In: *Methods in Cell Biology*. Academic Press Inc., pp. 325–366.

3. Seppa H, Grotendorst G, Seppa S, Schiffmann E, Martin GR. Platelet-derived growth factor in chemotactic for fibroblasts. *J Cell Biol* 1982; 92: 584–588.

4. Vannella KM, Wynn TA. Mechanisms of Organ Injury and Repair by Macrophages. *Annu Rev Physiol* 2017; 79: 593–617.

5. Bonyadi MR, Michalewicz Z. Particle swarm optimization for single objective continuous space problems: A review. *Evol Comput* 2017; 25: 1–54.

6. Zhang Y, Wang S, Ji G. A Comprehensive Survey on Particle Swarm Optimization Algorithm and Its Applications. *Math Probl Eng*; 2015. Epub ahead of print 2015. DOI: 10.1155/2015/931256.

7. Zhou X, Franklin RA, Adler M, Jacox JB, Bailis W, Shyer JA, et al. Circuit Design Features of a Stable Two-Cell System. *Cell* 2018; 172: 744-757.e17.

8. Adler M, Mayo A, Zhou X, Franklin RA, Meizlish ML, Medzhitov R, et al. Principles of Cell Circuits for Tissue Repair and Fibrosis. *iScience* 2020; 23: 100841.

9. Witte MB, Barbul A. General principles of wound healing. *Surgical Clinics of North America* 1997; 77: 509–528.

10. Jun J Il, Lau LF. The matricellular protein CCN1 induces fibroblast senescence and restricts fibrosis in cutaneous wound healing. *Nat Cell Biol* 2010; 12: 676–685.

11. McAndrews KM, Miyake T, Ehsanipour EA, Kelly PJ, Becker LM, McGrail DJ, et al. Dermal αSMA+ myofibroblasts orchestrate skin wound repair via β1 integrin and independent of type I collagen production. *EMBO J* 2022; 41: e109470.

12. Bain MA, Thibodeaux KT, Speyrer MS, Carlson E, Koullias GJ. Effect of Native Type I Collagen with Polyhexamethylene Biguanide Antimicrobial on Wounds: Interim Registry Results. *Plast Reconstr Surg Glob Open* 2019; 7: E2251.

13. Urciuolo F, Passariello R, Imparato G, Casale C, Netti PA. Bioengineered Wound Healing Skin Models: The Role of Immune Response and Endogenous ECM to Fully Replicate the Dynamic of Scar Tissue Formation In Vitro. *Bioengineering 2022, Vol 9, Page 233* 2022; 9: 233.

14. Ankit Rohatgi. WebPlotDigitizer, https://automeris.io/WebPlotDigitizer (2022).

15. Fortuna I, Perrone GC, Krug MS, Susin E, Belmonte JM, Thomas GL, et al. CompuCell3D Simulations Reproduce Mesenchymal Cell Migration on Flat Substrates. *Biophys J* 2020; 118: 2801–2815.

16. Shabestani Monfared G, Ertl P, Rothbauer M. An on-chip wound healing assay fabricated by xurography for evaluation of dermal fibroblast cell migration and wound closure. *Sci Rep* 2020; 10: 16192.

17. Haugh JM. Deterministic model of dermal wound invasion incorporating receptor-mediated signal transduction and spatial gradient sensing. *Biophys J* 2006; 90: 2297–2308.

18. Kihara T, Ito J, Miyake J. Measurement of Biomolecular Diffusion in Extracellular Matrix Condensed by Fibroblasts Using Fluorescence Correlation Spectroscopy. *PLoS One* 2013; 8: 82382.

19. Collier IE, Legant W, Marmer B, Lubman O, Saffarian S, Wakatsuki T, et al. Diffusion of MMPs on the surface of collagen fibrils: The mobile cell surface - collagen substratum interface. *PLoS One*; 6. Epub ahead of print 1 September 2011. DOI: 10.1371/journal.pone.0024029.

20. Saitou T, Rouzimaimaiti M, Koshikawa N, Seiki M, Ichikawa K, Suzuki T. Mathematical modeling of invadopodia formation. *J Theor Biol* 2012; 298: 138–146.

21. Uekita T, Itoh Y, Yana I, Ohno H, Seiki M. Cytoplasmic tail–dependent internalization of membrane-type 1 matrix metalloproteinase is important for its invasion-promoting activity. *J Cell Biol* 2001; 155: 1345.

22. Li W, You L, Schaffler MB, Wang L. The dependency of solute diffusion on molecular weight and shape in intact bone. *Bone* 2009; 45: 1017.

23. Cochran BH. The Molecular Action of Platelet-Derived Growth Factor. *Adv Cancer Res* 1985; 45: 183–216.

24. Hornbeck P V., Zhang B, Murray B, Kornhauser JM, Latham V, Skrzypek E. PhosphoSitePlus, 2014: mutations, PTMs and recalibrations. *Nucleic Acids Res* 2015; 43: D512–D520.

25. Freitas Robert A. *Nanomedicine Volume I: Basic Capabilities*. Landes Bioscience, 1999.

26. Volk SW, Wang Y, Mauldin EA, Liechty KW, Adams SL. Diminished Type III Collagen Promotes Myofibroblast Differentiation and Increases Scar Deposition in Cutaneous Wound Healing. *Cells Tissues Organs* 2011; 194: 25–37.

27. Li JF, Lowengrub J. The effects of cell compressibility, motility and contact inhibition on the growth of tumor cell clusters using the Cellular Potts Model. *J Theor Biol* 2014; 343: 79–91.

28. Teo YV, Rattanavirotkul N, Olova N, Salzano A, Quintanilla A, Tarrats N, et al. Notch Signaling Mediates Secondary Senescence. *Cell Rep* 2019; 27: 997-1007.e5.

29. Hoare M, Ito Y, Kang TW, Weekes MP, Matheson NJ, Patten DA, et al. NOTCH1 mediates a switch between two distinct secretomes during senescence. *Nat Cell Biol* 2016; 18: 979–992.

30. Kato K, Logsdon NJ, Shin YJ, Palumbo S, Knox A, Irish JD, et al. Impaired myofibroblast dedifferentiation contributes to nonresolving fibrosis in aging. *Am J Respir Cell Mol Biol* 2020; 62: 633–644.
